# Assessment of quality of life, psychosocial, and epidemiological aspects in patients diagnosed with tuberculous uveitis

**DOI:** 10.5935/0004-2749.2023-0042

**Published:** 2024-03-27

**Authors:** Luci Meire P. Silva, Tiago Eugênio Faria e Arantes, Aristófanes Canamary Jr, Yuslay Fernández Zamora, Luciana Peixoto S. Finamor, Ester Abigail Martins, Ricardo Pedro Casaroli-Marano, Cristina Muccioli

**Affiliations:** 1 Department of Ophthalmology and Visual Sciences, Hospital São Paulo, Universidade Federal de São Paulo, São Paulo, SP, Brazil; 2 Department of Surgery, School of Medicine & Hospital Clinic de Barcelona, University of Barcelona, Barcelona, Spain

**Keywords:** Tuberculosis, ocular, Quality of life, Uveitis, Anxiety, Depression, Surveys and questionnaires

## Abstract

**Purpose:**

To assess the quality of life in patients diagnosed as having tuberculous uveitis and its association with sociodemographic, clinical, and psychosocial aspects.

**Method:**

By conducting standardized interviews, clinical and demographic data were collected using a measure developed in this study. This measure was applied in addition to other measures, namely SF-12, Hospital Anxiety and Depression Scale, and NEI-VFQ-39, which were used to assess health-related quality of life, anxiety and depression symptoms, and visual functioning.

**Results:**

The study included 34 patients [mean age: 46.5 ± 15.1 years, female patients: 21 (61.8%)]. The mean of the VFQ-39 score was 74.5 ± 16.6 and that of SF-12 physical and mental component scores were 45.8 ± 10.1 and 51.6 ± 7.5, respectively, for the health-related quality of life. Anxiety symptoms were the most prevalent compared with depression symptoms and were found in 35.3% of the participants.

**Conclusion:**

Tuberculous uveitis affects several scales of quality of life, thereby affecting a population economically active with a social, psychological, and economic burden.

## INTRODUCTION

Visual impairment affects approximately 2.2 billion people globally^(^1^)^, impacting the physical and mental health of individuals and society, as it affects personal aspects as well as financial, social, and psychological aspects. It is an enormous global financial burden because of the annual costs of disability-associated losses. Adults with visual impairments often have lower rates of labor force participation and productivity^(1,^2^)^.

Uveitis is a group of inflammatory eye diseases mainly affecting the uveal tract, and adjacent structures. Sociodemographic aspects, geographic origin, and life habits can influence uveitis development^(^2^,^3^)^. Silva et al. reported that more than 50% of uveitis patients develop related complications, and up to 35% of patients develop severe visual impairment. Most studies have considered uveitis as a crucial cause of visual impairment secondary to functional and anatomical complications^(^4^)^. Uveitis is a chronic condition highly prevalent in young adults that exhibits a high incidence of relapses and possibly leads to visual impairment. The disease is associated with a high social cost owing to loss of productivity and, consequently, has a considerable socioeconomic impact on the entire community^(^2^,^5^)^. Moreover, long-term treatment for uveitis can cause mood swings, and fear of recurrence can lead to increased stress levels even with latent uveitis. These factors affect the quality of life (QoL) and productivity of these patients, thereby indicating a greater need of health care and its specialized services^(^6^)^.

Tuberculosis (TB) is the most prevalent contagious disease worldwide. One-third of the world population is estimated to be infected by TB. Globally, approximately 10 million people developed TB and 1.2 million died from the disease in 2019, although TB is the leading cause of preventable death. In 1993, the World Health Organization (WHO) declared TB a global public health emergency. Brazil is among the 30 countries with a high burden of TB and TB-HIV co-infection, being considered a priority for disease control by the WHO^(7)^.

TB mainly affects the lungs, but other bodily organs, such as the eye, can be also affected. The prevalence of extra pulmonary TB is 20% and that of ocular tuberculosis ranges from 3.3% to 5.22%, with posterior uveitis being the main ocular manifestation. Uveitis is thus a serious disease with a long recurrent course that often significantly decreases the visual functions and QoL of patients^(8-^11^)^.

Ocular manifestations such as tuberculous uveitis (TBU) is common in endemic regions. TBU represents one of the most challenging eye infectious diseases for uveitis experts. It typically affects many eye structures, mimicking inflammatory diseases and making its diagnosis difficult. In such cases, mostly a presumptive diagnosis of TB-related ocular inflammation is made ^(10,^11^)^. Delayed diagnosis or delayed treatment of TBU can cause severe visual impairment, thereby impacting the patient’s QoL on several domains of physical and mental health^(^9^)^.

The present study evaluates the QoL of individuals diagnosed as having TBU and its association with sociodemographic, clinic, and psychosocial aspects.

## METHODS

This observational, analytic, and cross-sectional cohort study included 34 patients who were diagnosed as having presumed TBU and being followed up at the Uveitis Sector of the Department of Ophthalmology at Hospital São Paulo-Federal University of São Paulo (UNIFESP). They were also part of the cohort included in other clinical studies, whose results have been published^(^9^,^11^)^. This study included patients who were under anti-tuberculosis therapy (ATT) and accepted to participate. All patients were treated with rifampicin, isoniazid, pyrazinamide, and ethambutol during the first 2 months, followed by rifampicin and isoniazid for the remaining 7 months. Each patient was interviewed in one visit, and the interview lasted approximately 40 min. The questionnaires were applied between the 3^rd^ and 9^th^ month after the ATT treatment was started.

### Ethical statements

This project was approved by the Ethics Committee for Clinical Research under number #CAAE 74600417.9.0000.5505. It was conducted in accordance with the Helsinki Declaration as well as with Resolution 466/12 of the National Health Council as defined under the Brazilian law^(12)^. The patients were fully informed about the objectives of the study and their informed consent was obtained.

### Data collection and criteria

The study inclusion criteria were as follows: (a) both genders aged more than 18 years; (b) ocular manifestations suggestive of TBU; (c) PPD skin test ≥10 mm; (d) positive IGRA (according to standard criteria); (e) participated in the TBU clinical study cohort and were under ATT; (f) can respond to the study interview and questionnaires; and (g) provided informed consent for participation.

During the interview, four data collection forms were completed by trained professionals. Of them, three were standardized forms [Short Form Health Survey (SF-12^®^), Hospital Anxiety and Depression Scale (HADS), and National Eye Institute-Visual Functioning Questionnaire-25 (NEI-VFQ-25)] and the fourth one was a form specifically developed by the investigators for collecting clinical, epidemiological, and sociodemographic data. To prevent the impact of disease-specific questions on the responses to general health questions, the generic forms (SF-12^®^ and HADS) were applied before the implementation of the disease-specific forms (NEI-VFQ-25 and study form) in that order: SF-12, HADS, NEI-VFQ-25, and questionnaire developed for the study.

### Sociodemographic and clinical data questionnaires

Personal data were collected through the specially developed questionnaire, which included the following questions: (a) Sociodemographic information: Age, ethnicity, gender, education level, monthly family income, marital status, current housing situation, people sharing same habitation, and professional occupation; (b) Clinical and epidemiological information about TB: ocular manifestation of TBU, laterality of disease, visual status, previous and current treatments, duration and discomfort with ATT, discrimination due to the disease, and leave from work; (c) Ocular and non-ocular comorbidities: type and concurrent treatments; and (d) Other additional information: illicit drug user and consumption of cigarettes or alcoholic beverages.

We applied SF12^®^ Health Survey, a concise version of SF-36^®(^13^)^. The physical component summary (PCS) and the mental component summary (MCS) of this questionnaire were previously validated in our scientific community^(^14^)^. We here considered the time range for memories in the last 4 weeks. HADS^(^15^)^, which has been translated and validated in many languages, including Portuguese, was used, and it has 14 items. Finally, we used the expanded form of NEI-VFQ-25 that includes 39 items (VFQ-39)^(^16^)^ to assess the QoL of individuals with chronic eye diseases through its all domains. This scale has been validated and translated into Portuguese^(^17^)^.

### Data analysis

Statistical analyses were performed using SPSS 20.0 for Windows (IBM SPSS Statistics; Chicago, IL, USA). Continuous variables were expressed as the mean ± standard deviation (SD) and range, while categorical variables were expressed as absolute and relative frequencies. Mann-Whitney U test and Kruskal-Wallis test were used to compare differences between groups. P<0.05 was considered statistically significant for all statistical tests.

## RESULTS

Data from 34 patients treated for TBU at the outpatient uveitis clinic of Hospital São Paulo-UNIFESP were collected. The mean age of the cohort was 46.5 ± 15.1 years, with 22 patients being females (61.8%). More than 90% of the patients were living with spouses and/or family, 55.9% of them were married, and 51.5% declared a familial income of $ 197- $ 590 (USD). Twenty-one patients (61.8%) had bilateral TBU involvement, while 13 (38.2%) had unilateral TBU involvement. Posterior uveitis was the most common type of manifestation (n=17, 50.0%), followed by pan uveitis (n=6, 17.6%) and anterior uveitis (n=4, 11.8%). Best corrected visual acuity in the better seeing eye was normal (0-0.4 logMAR) in 31 patients (91.2%), whereas 3 participants (8.8%) presented a low vision (0.48-0.9 logMAR). All patients were receiving ATT (n=34, 100.0%). Seven patients (20.6%) were referred to previous treatment for TB, and 12 patients (35.3%) were using a systemic corticosteroid and/or a corticosteroid-sparing agent for uveitis treatment. Table 1 presents the characteristics of studied participants.

**Table 1:** Sociodemographic and clinical characteristics of subjects with ocular tuberculosis (n=34)

Variable	p-value
Age (years)	
Mean ± SD (range)	46.5 ± 15.1 (20 - 72)
Age category, n (%)	
<40 years	12 (35.3)
40 - 60 years	16 (47.1)
>60 years	6 (17.6)
Ethnicity, n (%)	
Caucasian	19 (55.9)
African-Brazilian	7 (20.6)
Multiracial	8 (23.5)
Gender, n (%)	
Female	21 (61.8)
Male	13 (38.2)
Educational level, n (%)	
Illiterate and incomplete elementary school	3 (8.8)
Complete elementary school	5 (14.7)
Complete high school	18 (52.9)
Complete graduation	8 (23.5)
Monthly household income, n (%)^a^	
<197 USD	7 (21.2)
From 197 to 590 USD	17 (51.5)
>590 USD	9 (27.3)
Marital status, n (%)	
Married	19 (55.9)
Single	10 (29.4)
Divorced	4 (11.8)
Widow	1 (2.9)
Current housing situation, n (%)	
With family	32 (94.1)
Alone	2 (5.9)
People sharing same habitation, n (%)	
Alone	2 (5.9)
1 - 3 individuals	22 (64.7)
>4 individuals	10 (29.4)
Employment status, n (%)	
Unemployed	9 (26.5)
Employed	19 (55.9)
Retired	3 (8.8)
Temporarily away from work	2 (5.8)
Housewife	1 (2.9)
Ocular manifestation of TBU, n (%)	
Anterior uveitis	4 (11.8)
Intermediate uveitis	3 (8.8)
Posterior uveitis	17 (50.0)
Pan uveitis	6 (17.6)
Other^b^	4 (11.8)
Laterality, n (%)	
Unilateral	13 (38.2)
Bilateral	21 (61.8)
Visual status, n (%)	
Normal vision (0-0.4 logMAR in the better seeing eye)	31 (91,2)
Low vision (0.48-0.9 logMAR in the better seeing eye)	3 (8.8)

a Monthly household income was not informed by 1 subject.

b Scleritis (n=1), keratoscleritis (n=1), sclerouvetis (n=1), and keratitis (n=1)

### General Health-Related QoL (SF-12v2)

The studied sample had a score of >45 for both PCS and MCS (PCS: 45.8 ± 10.1 and MCS: 51.6 ± 7.5). One item exhibited a score of <45 that is, social functioning (38.7 ± 8.9) (Table 2).

**Table 2:** 12-item Short-Form Health Survey (SF-12v2) scores for the eight domains, and the physical and mental component summary of subjects with ocular tuberculosis (n=34 subjects)

Variable	Mean ± SD (range)
Physical Functioning (PF)	46.7 ± 8.9 (26.4 - 56.47)
Role Physical (RP)	46.3 ± 12.3 (20.3 - 57.1)
Bodily Pain (BP)	48.5 ± 11.2 (21.8 - 57.4)
General Health (GH)	52.5 ± 12.9 (18.9 - 62.0)
Vitality (VIT)	48.0 ± 10.9 (27.6 - 67.9)
Social Functioning (SF)	38.7 ± 8.9 (26.3 - 56.6)
Role Emotional (RE)	52.1 ± 7.2 (33.7 - 56.1)
Mental Health (MH)	55.9 ± 11.2 (28.0 - 64.5)
Physical Component Summary (PCS)	45.8 ± 10.1 (14.3 - 57.9)
Mental Component Summary (MCS)	51.6 ± 7.5 (33.3 - 64.0)

Clinical and sociodemographic characteristics were significantly associated with some domains (p<0.05): (a) older age was associated with the reduced role physical score; (b) lower monthly household income was associated with the reduced role emotional score; (c) patients with a unilateral disease had lower mental health scores; (d) undergoing previous TB treatment was associated with a lower social functioning score; (e) patients experiencing prejudice because of TB had lower bodily pain scores; and (f) patients receiving concurrent treatment for uveitis had lower mental health scores (Supplemental data - S1).

**Table 3:** Hospital Anxiety and Depression Scale (HADS) scores of subjects with ocular tuberculosis (n=34)

Anxiety	
Mean ± SD (range)	6.5 ± 3.3 (0 - 13)
Classification, n (%)	
Normal (0 - 7)	22 (64.7)
Mild (8 - 10)	7 (20.6)
Moderate (11 - 14)	5 (14.7)
**Depression**	
Mean ± SD (range)	4.5 ± 4.0 (0 - 20)
Classification, n (%)	
Normal (0 - 7)	28 (82.4)
Mild (8 - 10)	4 (11.8)
Moderate (11 - 14)	1 (2.9)
Severe (15 - 21)	1 (2.9)

### Hospital Anxiety and Depression Scale

Twelve (35.3%) and 6 (17.6%) patients had anxiety and depression symptoms ranking from mild to severe, respectively. The mean scores for anxiety and depression were 6.5 ± 3.3 and 4.5 ± 4.0, respectively (Table 3). Monthly household income was significantly (p<0.05) associated with anxiety and depression scores. Undergoing previous TB treatment was also significantly (p<0.05) associated with depression scores (Supplemental data - S2).

**Table 4:** National Eye Institute’s 39-Item Visual Function Questionnaire (NEI VFQ-39) scores of subjects with ocular tuberculosis (n=34)

Variable	Mean ± SD (range)
General health	64.8 ± 20.5 (20.0 - 100.0)
General Vision	67.6 ± 12.3 (45.0 - 100.0)
Ocular Pain	74.6 ± 22.1 (25.0 - 100.0)
Near Activities	73.6 ± 20.9 (20.8 - 100.0)
Distance Activities	71.4 ± 22.2 (25.0 - 100.0)
Social Functioning	89.5 ± 15.5 (41.7 - 100.0)
Mental Health	61.8 ± 22.7 (20.0 - 100.0)
Role Difficulties	70.8 ± 27.1 (12.5 - 100.0)
Dependency	77.0 ± 25.1 (12.5 - 100.0)
Driving	63.6 ± 29.9 (0.0 - 100.0)
Color Vision	93.9 ± 16.6 (50.0 - 100.0)
Peripheral Vision	67.6 ± 30.5 (25.0 - 100.0)
Mean composite score	74.5 ± 16.6 (36.7 - 97.1)

### National Eye Institute’s 39-Item Visual Function Questionnaire

The global composite score was 74.5 ± 16.6 (Table 4). NEI-VFQ-39 categories scores per selected sociodemographic and clinical characteristics. Low vision was significantly associated with a higher general health score, but it was also significantly associated with lower scores of general vision, near activities, distance activities, mental health, role difficulties, dependency, and color vision, and lower mean composite score. Significant associations were also observed between peripheral vision and age; general vision and monthly household income; ocular manifestation with social functioning and peripheral vision; experiencing prejudice because of TB with general health, near activities, distance activities, color vision, and mean composite score; concurrent uveitis treatment with general health, general vision, near activities, distance activities, social functioning, mental health, role difficulties, dependency, peripheral vision, and mean composite score (Supplemental data - S3).

## DISCUSSION

This study assessed QoL in patients diagnosed as having TBU and its associations with sociodemographic, clinical, and psychological aspects. Domain scores of general health (SF-12v2) and scores of the vision questionnaire (NEI-VFQ-39) and psychological scale (HADS) were affected compared with some variables (age, educational level, monthly household income, disease laterality, and concurrent uveitis treatment); this is similar to the findings of other studies involving uveitis patients.

Our cohort involved a young population diagnosed as having TBU, with the prevalence of uveitis being higher in female participants (61.8%). Several studies on uveitis have reported a prevalence of 56.8%-64.4% in women^(^2^,^5^,^9^,^18^,^19^)^. Although our study focused on a specific infectious uveitis etiology, it included patients of an age range similar to that observed in other uveitis studies, revealing that uveitis affecting an adult working age population can have social and economic implications directly to the individual because this condition can affect employment status and consequently family income^(^2^,^7^,^19^)^. No gender-related differences were noted with the use of the questionnaire or scale, although some studies have reported that women possibly take more care of and pay more attention to their health than men and that gender is associated with lower scores in general health questionnaires^(^20^)^.

Regarding general health-related QoL (SF-12), our study sample had a score within normal ranges (above 45) for both PCS and MCS. However, the social functioning item had a score of <40, which indicated an impaired function. Our study population had higher PCS and MCS scores compared with that of a study on uveitis of both infectious and non-infectious origin^(^2^,^19^)^. The mentioned study had the largest number of noninfectious uveitis cases and lower general health scores for this condition, which may be justified by the chronicity of the disease and its recurrence, and prolonged use of medications such as corticosteroids or immunosuppressants. In our cohort, significantly lower scores were noted in patients undergoing previous TB treatment, which indicated that the use of medications was negatively correlated to scores of general health questionnaires, as previously reported.

Normative data from SF-36 were used for comparing QoL scores in the south Brazilian population, and these data are used for comparing groups in the absence of a gold standard^(^21^)^. Different from the results of the present study, female participants among the general Brazilian population studied had lower QoL scores on the general health questionnaire. Similarly, other variables, such as low education level, and some characteristics of the chronic medical condition, such as previous and concurrent treatment and (also) experiencing prejudice with diseases, contributed to a lower QoL score on the questionnaire. In the previous study involving normative data, age also negatively affected general health, similar to that in the present study, although both studies included patients belonging to a different age range. Our study cohort included patients aged >60 years, while the previous study included participants aged between 30 and 44 years^(^21^)^. Similar to another study, we noted that older age was associated with the reduced role physical score. The other study observed statistical significance for the same domain in SF-36 in elderly people who had reduced visual acuity from uveitis of diverse etiologies ranging from mild to severe^(^22^)^.

Lower economic status was significantly associated with the reduced role emotional score on SF-12, which is contradictory to the results of another study in which income influenced physical functioning. In that study, 26.3% of the population had incomplete elementary school education or were illiterate compared with only approximately 9% of our study sample with incomplete elementary school education or who were illiterate^(^2^)^.

Patients with intermediate uveitis and pan uveitis had lower scores on the NEI-VFQ-39 questionnaire, possibly because of the worse visual acuity related to these clinical presentations of TBU. Patients in the current treatment had higher scores on the vision questionnaire. This may be because their vision improved and they felt hopeful that they were receiving appropriate treatment, even if it required prolonged treatment and taking a few pills daily. In other studies, low visual acuity in uveitis patients was associated with lower levels of vision--related QoL in the specific questionnaires. This finding is similar to our findings that the normal vision population have higher scores in most vision-related domains on the VFQ-25 questionnaire^(^23^)^. However, the overall score (GCS - VFQ-25) of our population was lower than that of a study sample of healthy age-matched people^(^24^)^.

On comparing with the scores (GCS - VFQ-25) for other ophthalmic diseases, we noted that the score for TBU (74.5) was higher than that for severe glaucoma (58.7) and age-related macular degeneration (72.2) and lower than that for ocular toxoplasmosis (75.3) and diabetic retinopathy (79.9). This demonstrates that the two main causes of infectious uveitis in Brazil (toxoplasmosis and TB), and probably in the world, have similar scores. Therefore, these two causes can affect the visual QoL in a similar manner. Moreover, they presented lower scores than those presented by systemic diseases, such as diabetes. Thus, TBU has a major impact on an individual’s health^(6,^25^,^26^)^.

Our results also showed that although most patients presented anxiety and depression levels, considered as normal according to HADS, 35.3% and 17.6% of these patients were classified to present abnormal levels of anxiety and depression, respectively. These percentages are similar to the results observed in a recent meta--analysis^(^27^)^. In the meta-analysis of 12 observational studies involving 874 uveitis patients, behavioral changes were noted in 39% and 17% patients, respectively, with anxiety and depression symptoms. Similar results (anxiety=38%; depression=19%) were noted previously^(6)^, but in a smaller population (n=81) with ocular toxoplasmosis. Our sample exhibited a rate of anxiety twice higher than that of depression, which follows the trend that the overall prevalence of anxiety is usually higher than that of depression^(27)^. In our cohort, we also observed an association between variables, such as low family income, and anxiety and depression. Furthermore, the absence of previous treatment influenced depressive symptoms. HADS is a self-reported questionnaire, and not a psychiatric assessment, and the relationship between psychological measures and visual problems may be delicate.

Psychological aspects of uveitis patients may be affected, and this could be particularly related to vision restriction that leads to difficulties in daily activities. Side effects of medications and disease recurrence are some other factors influencing the psychological aspects of the uveitis population^(27,^28^)^. Several studies have reported that the incidence of psychiatric disorders in TBU patients is higher than that in the general population. We believe that this is probably due to poor prognosis, fear of vision loss, prolonged treatment, concurrent systemic disease, social acceptance, and stigmatization for the disease, which results in anxiety and depression^(29,^30^)^.

Our study has some limitations. QoL is inevitably a subjective tool that can be affected in different ways. Lower educational levels lead to misunderstandings, thereby contributing to response bias. These biases were reduced by clarifying the doubts of the responders during the interviews and ensuring that the interviews were conducted under sufficient privacy in a calm environment. Moreover, data were collected using a standardized method and by a trained team. Additionally, a poor emotional and physical health can negatively affect responses. To minimize the negative impact on answers, we always applied the generic forms before applying the disease-specific forms. Although objective measures are available, a gold standard for assessing QoL is lacking. This is a limitation of our study and could be an interesting future research topic. The present study included a small number of participants because our cohort was designed as part of the main TBU clinical and translational study. All participants enrolled in the former study, who were receiving ATT, were invited to participate in the present study. However, not all accepted to participate in the interviews, which resulted in a loss of the study population. Thus, the sample size was not calculated and was limited by patient availability, which could have restricted the study power.

Currently, despite the lack of other studies related to QoL of TBU patients, the disease is known to deeply affect several aspects of the life of such patients.

TBU has an impact on several aspects of QoL of the affected patients. It thus poses a social, psychological, and economic burden on TBU patients. Factors such as age, monthly household income, educational level, prolonged and concurrent treatments, social acceptance, and stigmatization for the disease, as well as visual impairment secondary to eye disease, can negatively influence the QoL of these patients.

## Appendix

Supplemental Table S1 Short-Form (SF-12) Health Survey scores among subjects with ocular tuberculosis (unless otherwise stated, n=34 total subjects)

**Table:**

	Physical Functioning	Role Physical	Bodily Pain	General Health	Vitality	Role Emotional	Social Functioning	Mental Health	Physical Component Summary	Mental Component Summary
Gender, n (%)										
Female, 21 (61.8)	46.6 ± 9.1	47.1 ± 12.0	50.4 ± 8.0	51.2 ± 12.3	48.7 ± 11.4	51.8 ± 7.5	37.8 ± 8.0	54.1 ± 12.5	46.8 ± 7.8	50.3 ± 8.3
Male, 13 (38.2)	46.9 ± 8.8	45.1 ± 13.2	45.3 ± 14.9	54.5 ± 14.2	47.0 ± 10.4	52.6 ± 7.1	40.3 ± 10.5	58.9 ± 8.4	44.1 ± 13.2	53.8 ± 5.7
Age, n (%)										
<40 years, 12 (35.3)	48.9 ± 6.9	**49.5 ± 8.6**	49.8 ± 9.6	53.0 ± 14.4	46.9 ± 10.9	52.3 ± 7.3	36.4 ± 8.6	54.9 ± 11.2	48.5 ± 9.2	49.3 ± 7.9
40 to 60 years, 16 (47.1)	44.9 ± 10.4	**48.5 ± 12.3**	47.9 ± 12.7	55.3 ± 11.0	50.9 ±11.5	51.9 ± 8.0	41.4 ± 9.0	57.3 ± 10.9	46.1 ± 10.8	53.7 ± 6.7
>60 years. 6 (17.6)	47.1 ± 8.3	**34.1 ± 12.7**	47.2 ± 11.6	44.0 ± 13.1	42.7 ± 8.4	52.4 ± 5.8	36.4 ± 9.0	54.4 ± 13.7	39.3 ± 8.3	50.7 ± 8.44
Ethnicity, n (%)										
Caucasian, 19 (55.9)	46.3 ± 8.0	45.5 ± 12.6	47.0 ± 11.8	51.8 ± 14.1	48.3 ± 12.3	52.0 ± 7.6	36.9 ± 7.9	57.2 ± 9.2	44.4 ± 11.4	52.1 ± 6.55
African-brazilian (7 (20.6)	44.8 ± 10.1	48.0 ± 10.9	48.7 ± 10.9	48.1 ± 13.5	43.4 ± 7.9	49.7 ± 8.8	42.1 ± 7.9	52.4 ± 13.6	45.6 ± 9.3	48.9 ± 9.6
Multiracial, 8 (23.5)	49.5 ± 10.2	46.8 ± 12.5	51.7 ± 10.7	57.9 ± 8.0	51.2 ± 9.2	54.7 ± 3.9	40.2 ± 12.0	56.2 ± 14.2	49.0 ± 7.5	53.0 ± 8.2
Educational level, n (%)										
Illiterate and incomplete elementary school, 3 (8.8)	36.4 ± 10.8	32.6 ± 14.1	37.1 ± 17.6	36.8 ± 12.4	**34.3 ± 11.6**	48.6 ± 6.5	46.5 ± 10.1	50.3 ± 19.6	30.5 ± 16.8	51.7 ± 13.0
Complete elementary school, 5 (14.7)	51.3 ± 7.7	44.3 ± 17.9	52.3 ± 7.2	53.4 ± 14.1	**49.8 ± 13.1**	53.8 ± 5.0	42.4 ± 11.5	60.1 ± 5.4	47.0 ± 16.7	55.0 ± 5.8
Complete high school, 18 (52.9)	47.6 ± 8.8	50.5 ± 9.9	50.6 ± 9.2	55.4 ± 9.2	**52.2 ± 9.3**	52.3 ± .7.7	36.9 ± 5.4	55.4 ± 11.7	49.3 ± 6.7	50.1 ± 6.7
Complete graduation, 8 (23.5)	45.7 ± 7.3	43.3 ± 9.8	45.3 ± 13.3	51.2 ± 17.3	**42.7 ± 7.6**	51.9 ± 8.3	37.6 ± 12.6	56.2 ± 10.3	42.8 ± 9.1	51.0 ± 8.6
Monthly household incomes^a^, n (%)										
<197USD, 7 (21.2)	39.3 ± 11.9	38.7 ± 16.0	45.1 ± 10.9	43.5 ± 13.5	46.3 ± 12.2	**44.9 ± 9.1**	40.7 ± 9.8	51.5 ± 11.9	38.9 ± 12.9	49.6 ± 8.3
197 to 590USD, 17 (51.5)	49.6 ± 6.8	49.0 ± 10.7	49.3 ± 10.4	56.3 ± 9.4	50.1 ± 12.1	**54.8 ± 3.7**	39.9 ± 6.1	57.0 ± 11.1	49.5 ± 8.0	53.0 ± 6.9
>590USD, 9 (27.3)	46.9 ± 7.7	48.0 ± 11.3	48.4 ± 13.9	52.4 ± 16.6	45.5 ± 8.4	**52.4 ± 7.9**	63.4 ± 12.4	59.1 ± 10.3	45.6 ± 10.6	51.8 ± 8.1
	Physical Functioning	Role Physical	Bodily Pain	General Health	Vitality	Role Emotional	Social Functioning	Mental Health	Physical Component Summary	Mental Component Summary
Marital status, n (%)										
Married, 19 (55.9)	47.9 ± 9.2	46.5 ± 12.4	46.2 ± 12.7	53.5 ± 13.7	48.3 ± 11.8	52.0 ± 7.6	38.5 ± 7.9	58.1 ± 10.2	45.4 ± 11.7	52.5 ± 7.9
Single, 10 (29.4)	45.7 ± 8.6	46.1 ± 11.3	50.8 ± 9.6	53.4 ± 9.9	47.7 ± 11.6	53.8 ± 4.7	40.4 ± 9.7	50.5 ± 13.8	47.1 ± 7.4	50.1 ± 7.4
Divorced. 4 (11.8)	47.9 ± 6.1	43.4 ± 17.6	53.6 ± 7.6	51.2 ± 15.2	47.7 ± 8.2	53.3 ± 5.6	36.4 ± 14.3	60.0 ± 5.8	45.4 ± 11.2	53.0 ± 6.3
Widow. 1 (2.9)	30.7	57.2	47.2	29.6	47.7	33.7	63.4	52.3	41.0	44.0
Current housing situation, n (%)										
With spouse or family, 32 (94,1)	46.4 ± 9.0	46.5 ± 12.2	48.2 ± 11.5	52.2 ± 13.2	47.4 ± 10.7	51.9 ± 7.4	38.3 ± 9.0	55.8 ±± 11.6	45.6 ± 10.3	51.3 ± 7.6
Alone, 2 (5.9)	52.2 ± 6.1	43.4 ± 19.6	52.3 ± 7.2	56.6 ± 7.6	57.8 ± 14.2	56.1 ± 0.0	46.5 ± 0.0	58.4 ± 0.0	48.2 ± 8.8	57.6 ± 1.5
People sharing same habitation, n (%)										
Alone, 2 (5.9)	52.2 ± 6.1	43.4 ± 19.6	52.3 ± 7.2	56.6 ±7.6	57.8 ± 14.2	56.1 ± 0.0	46.5 ± 0.0	58.4 ± 0.0	48.2 ± 8.9	57.6 ± 1.5
1 - 3 individuals, 22 (64.7)	44.7 ± 9.6	44.2 ± 12.6	46.3 ± 11.9	50.7 ± 14.3	45.0 ± 9.4	50.5 ± 5.3	38.7 ± 9.1	55.1 ± 11.1	43.5 ± 10.7	50.7 ± 7.8
>4 individuals, 10 (39.4)	50.0 ± 6.5	51.6 ± 9.9	52.4 ± 9.6	55.5 ± 10.4	52.8 ± 11.9	55.0 ± 3.5	37.4 ± 8.8	57.2 ± 13.1	50.3 ± 7.7	52.4 ± 7.2
Employment status, n (%)										
Unemployed + housewife, 10 (29,4)	48.3 ± 8.2	45.2 ± 13.1	52.3 ± 5.4	51.2 ± 13.4	47.7 ± 8.2	52.7 ± 7.5	39.4 ± 9.6	57.8 ± 8.3	46.4 ± 7.5	52.4 ± 6.6
Employed, 19 (55.9)	46.5 ± 9.4	46.0 ± 12.9	45.1 ± 13.1	52.9 ± 13.1	48.3 ± 12.3	51.9 ± 6.7	40.1 ± 9.0	54.6 ± 12.4	44.9 ± 11.8	51.7 ± 7.8
Retired and away from work, 5 (14,6)	44.4 ± 9.3	49.8 ± 10.1	53.4 ± 9.1	53.4 ± 14.1	47.7 ± 12.3	51.6 ± 10.0	32.3 ± 5.5	57.2 ± 13.2	47.6 ± 8.9	49.8 ± 9.5
Ocular manifestation, n (%)										
Anterior uveitis, 4 (11,8)	42.5 ± 5.4	45.6 ± 13.8	49.8 ± 15.3	51.2 ± 15.2	47.7 ± 16.4	53.2 ± 5.6	36.4 ± 8.2	52.3 ±16.5	44.4 ± 11.5	50.6 ± 9.7
Intermediate uveitis, 3 (8.8)	42.1 ± 9.9	44.9 ± 5.3	47.2 ± 10.2	47.6 ± 16.5	44.4 ± 5.8	48.6 ± 12.9	39.7 ± 15.4	48.3 ± 12.7	44.0 ± 7.6	47.2 ± 12.6
Posterior uveitis, 17 (50,0)	48.1 ± 9.5	48.5 ± 11.5	48.7 ± 11.9	55.0 ± 12.6	48.3 ± 11.5	52.1 ± 6.7	39.9 ± 9.4	56.7 ± 11.6	47.7 ± 11.8	51.7 ± 7.6
Panuveitis, 6 (16.7)	49.3 ± 8.4	43.3 ± 15.1	48.1 ± 13.4	54.8 ± 8.8	51.1 ± 10.4	56.1 ± 0.0	39.7 ± 8.2	59.5 ± 9.8	44.8 ± 7.9	55.8 ± 2.8
Other, 4 (11.8)	44.6 ± 9.5	43.3 ± 17.6	47.2 ± 4.1	43.1 ± 16.2	45.2 ± 9.6	47.7 ± 10.7	33.8 ± 5.0	56.9 ± 5.8	41±7 ± 7.6	49.3 ± 6.0
Laterality, n (%)										
Unilateral, 13 (38.2)	45.2 ± 9.2	46.5 ± 10.5	48.0 ± 11.2	53.7 ± 11.8	48.5 ± 11.9	51.2 ± 7.3	39.5 ± 9.6	**49.5 ± 13.5**	46.9 ± 8.4	49.0 ± 9.9
Bilateral, 21 (61.8)	47.7 ± 8.7	46.2 ± 13.5	48.7 ± 11.5	51.7 ± 13.8	47.7 ± 10.5	52.3 ± 7.4	38.3 ± 8.8	**59.9 ± 7.4**	45.1 ± 11.2	53.2 ± 5.1
Visual status, n (%)										
Normal vision, 31 (91.2)	46.3 ± 8.8	45.9 ± 12.5	48.4 ± 11.4	52.2 ± 13.4	46.8 ± 10.2	52.5 ± 6.7	39.0 ± 9.4	55.7 ± 11.4	45.3 ± 10.3	51.6 ± 7.5
Low vision, 3 (8.8)	50.7 ± 9.9	51.0 ± 10.6	48.9 ± 10.6	54.8 ± 6.22	61.2 ± 11.6	48.6 ± 12.9	36.4 ± 0.0	52.4 ± 10.6	50.4 ± 7.8	52.2 ± 8.8
Previous TB treatment, n (%)										
Yes, 7 (20,6)	46.0 ± 6.9	44.0 ± 10.4	44.3 ± 14.1	48.1 ± 17.3	44.9 ± 15.0	49.7 ± 8.8	**32.0 ± 5.4**	52.4 ± 14.5	43.5 ± 11.4	47.2 ± 7.27
No, 27 (79,4)	46.9 ± 9.4	46.9 ± 12.9	49.5 ± 10.4	53.6 ± 11.7	48.9 ± 9.8	52.8 ± 6.8	**40.5 ± 9.0**	56.9 ± 10.4	46.4 ± 9.9	52.8 ± 7.3
Experienced prejudice because TB, n (%)										
Yes, 8 (23,5)	48.4 ± 9.3	43.3 ± 13.9	**43.4 ± 9.7**	51.2 ± 14.1	49.0 ± 10.0	47.7 ± 9.9	37.6 ± 10.0	52.3 ± 11.7	45.2 ± 10.3	48.3 ± 11.0
No, 26 (76,5)	46.2 ± 8.8	47.2 ± 11.9	**50.0 ± 11.4**	52.9 ± 12.8	47.7 ± 11.4	53.5 ± 5.7	39.1 ± 8.8	57.0 ± 11.1	45.9 ± 10.2	52.6 ± 6.0
Concurrent uveitis treatment, n (%)										
Yes, 12 (35,3)	43.9 ± 8.7	46.4 ± 12.3	46.8 ± 10.9	52.1 ± 11.7	51.9 ± 10.0	50.5 ± 8.9	35.5 ± 8.0	**51.3 ± 12.1**	45.5 ± 8.1	49.5 ± 7.6
No, 22 (64,7)	48.3 ± 8.8	46.3 ± 12.6	49.3 ± 11.5	52.7 ± 13.8	45.9 ± 11.0	53.0 ± 6.1	40.5 ± 9.2	**58.4 ± 10.1**	45.9 ± 11.2	52.7 ± 7.4

TB= tuberculosis. ^a^Monthly household income was not informed by one participant. Statistical analyses were performed using the Mann-Whittney U-Test (bold values denote statistical significance at p<0.05 level.

Supplemental Table S2 Hospital Anxiety and Depression Scale (HADS) scores among participants with ocular tuberculosis (unless otherwise stated, n=34 total participants)

**Table:**

	Anxiety	Depression
Gender,n(%)		
Female,21(61.8)	6.95 ± 3.5	4.2 ± 3.1
Male,13(38.2)	5.8 ± 3.1	5.0 ± 5.2
Age,n(%)		
<40years,12(35.3)	6.8 ± 3.3	4.3 ± 2.0
40to60years,16(47.1)	6.3 ± 3.5	4.8 ± 5.5
>60years,6(17.6)	6.5 ± 3.3	4.2 ± 2.4
Ethnicity,n(%)		
Caucasian,19(55.9)	6.4 ± 2.9	5.0 ± 4.4
African-Brazilian(7(20.6)	8.0 ± 2.6	3.7 ± 2.1
Multiracial,8(23.5)	5.4 ± 4.6	4.1 ± 4.4
Educationallevel,n(%)		
Illiterateandincompleteelementaryschool,3(8.8)	8.0 ± 1.7	9.0 ± 9.6
Completeelementaryschool,5(14.7)	2.2 ± 4.1	3.8 ± 2.9
Completehighschool,18(52.9)	6.4 ± 3.6	4.1 ± 3.4
Completegraduation,8(23.5)	6.9 ± 2.5	4.4 ± 2.5
Monthlyhouseholdincomes^a^,n(%)		
<197USD,7(21.2)	**9.4 ± 2.9**	**9.0 ± 6.1**
197to590USD,17(51.5)	**5.2 ± 2.8**	**2.9 ± 1.8**
>590USD,9(27.3)	**6.2 ± 3.0**	**4.0 ± 2.6**
Maritalstatus,n(%)		
Married,19(55.9)	5.8 ± 2.7	4.5 ± 4.6
Single,10(29.4)	7.7 ± 3.3	4.5 ± 3.3
Divorced.4(11.8)	5.3 ± 4.6	4.0 ± 3.4
Widow.1(2.9)	13.0	8.0
Currenthousingsituation,n(%)		
Withspouseorfamily,32(94,1)	6.5 ± 3.4	4.7 ± 4.0
Alone,2(5.9)	6.5 ± 0.7	1.5 ± 2.1
Peoplesharingsamehabitation,n(%)		
Alone,2(5.9)	6.5 ± 0.7	1.5 ± 2.1
1-3individuals,22(64.7)	6.7 ± 3.4	5.3 ± 4.6
>4individuals,10(39.4)	6.3 ± 3.7	3.4 ± 1.9
Employmentstatus,n(%)		
Unemployed+housewife,10(29,4)	6.6 ± 4.5	3.8 ± 2.9
Employed,19(55.9)	6.8 ± 2.7	4.9 ± 4.8
Retiredandawayfromwork,5(14,6)	5.2 ± 2.9	4.6 ± 2.3
Ocularmanifestation,n(%)		
Anterioruveitis,4(11,8)	6.3 ± 2.0	3.3 ± 2.4
Intermediateuveitis,3(8.8)	7.3 ± 2.3	4.7 ± 3.1
Posterioruveitis,17(50,0)	6.5 ± 3.7	4.9 ± 5.1
Panuveitis,6(16.7)	5.0 ± 2.2	3.3 ± 1.9
Other,4(11.8)	8.5 ± 4.4	537 ± 2.9
Laterality,n(%)		
Unilateral,13(38.2)	7.1 ± 3.2	4.3 ± 3.5
Bilateral,21(61.8)	6.1 ± 3.4	4.7 ± 4.3
Visualstatus,n(%)		
Normalvision,31(91.2)	6.7 ± 3.4	4.4 ± 4.1
Lowvision,3(8.8)	4.7 ± 2.1	5.3 ± 3.5
PreviousTBtreatment,n(%)		
Yes,7(20,6)	6.4 ± 3.5	**4.1 ± 4.3**
No,27(79,4)	6.9 ± 2.7	**6.1 ± 1.5**
ExperiencedprejudicebecauseTB,n(%)		
Yes,8(23,5)	6.2 ± 3.4	4.8 ± 3.2
No,26(76,5)	6.7 ± 3.4	4.4 ± 4.2
Concurrentuveitistreatment,n(%)		
Yes,12(35,3)	6.9 ± 3.1	5.5 ± 3.4
No,22(64,7)	6.3 ± 3.5	4.0 ± 4.2

TB= tuberculosis. ^a^Monthly household income was not informed by one participant. Statistical analyses were performed using the Mann-Whittney U-Test (bold values denote statistical significance at the p<0.05 level.

Supplemental Table 3 National Eye Institute Visual Function Questionnaire (NEI-VFQ-39) scores among participants with ocular tuberculosis (unless otherwise stated, n=34 total participants)

**Table:**

	General health	General Vision	Ocular Pain	Near Activities	Distance Activities	Social Functioning	Mental Health	Role Difficulties	Dependency	Driving^a^	Color Vision	Peripheral Vision	Mean composite score
Gender, n (%)													
Female, 21 (61.8)	65.4 **± 20.2**	69.5 **± 12.9**	73.8 **± 24.3**	76.0 **± 20.8**	74.5 **± 19.7**	89.3 **± 15.4**	65.5 **± 20.9**	77.4 **± 22.8**	83.0 **± 16.4**	54.2 **± 17.7**	92.9 **± 17.9**	64.3 **± 32.2**	76.6 **± 16.1**
Male, 13 (38.2)	63.8 ± 21.7	64.6 ± 10.9	75.9 ± 18.7	69.7 ± 21.2	66.4 ± 25.8	89.7 ± 16.4	55.8 ± 25.0	60.1 ± 30.1	67.3 ± 33.4	65.7 ± 32.4	95.8 ± 14.4	73.1 ± 27.9	71.0 ± 17.5
Age, n (%)													
≤40 years, 12 (35.3)	60.8 ± 23.0	68.8 ± 11.5	70.8 ± 23.4	80.8 ± 24.5	73.5 ± 24.6	92.4 ± 14.0	60.0 ± 23.3	66.7 ± 28.7	77 ± 25.6	75.0 ± 26.4	91.7 ± 19.5	**79.2 ± 25.7**	76.3 ± 17.1
40 to 60 years, 16 (47.1)	69.8 ± 17.6	67.2 ± 14.4	74.2 ± 23.0	68.8 ± 18.3	68.4 ± 22.7	86.5 ± 16.6	60.9 ± 24.4	69.1 ± 26.2	72.7 ± 26.3	46.7 ± 32.0	93.3 ± 17.6	**53.1 ± 31.5**	70.7 ± 17.9
>60 years. 6 (17.6)	59.2 ± 22.7	66.7 ± 8.7	83.3 ± 17.1	72.1 ± 18.6	75.3 ± 17.7	91.7 ± 16.7	67.5 ± 18.9	83.3 ± 27.3	87.5 ± 20.9	83.3 ± 0.0	100.0 ± 0.0	**83.3 ± 20.4**	81.0 ± 10.6
Ethnicity, n (%)													
Caucasian, 19 (55.9)	67.9 ± 21.1	68.9 ± 14.5	71.7 ± 22.8	69.9 ± 25.3	69.7 ± 27.5	88.2 ± 17.6	58.4 ± 28.2	65.1 ± 32.8	71.4 ± 30.4	61.5 ± 33.6	91.7 ± 19.2	71.5 ± 32.6	72.2 ± 20.9
African-Brazilian (7 (20.6)	57.1 ± 22.6	61.4 ± 9.4	76.8 ± 28.3	74.7 ± 8.5	78.7 ± 17.1	89.3 ± 15.0	62.8 ± 15.5	84.8 ± 13.4	80.4 ± 18.6	91	2.9 ± 18.9	71.4 ± 30.4	77.4 ± 10.6
Multiracial, 8 (23.5)	64.1 ± 17.6	70.0 ± 6.5	79.7 ± 14.8	81.4 ± 15.2	69.1 ± 7.7	92.7 ± 11.3	68.8 ± 9.2	71.9 ± 16.4	87.5 ± 8.2	58.3 ± 11.8	100.0 ± 0.0	56.3 ± 25.9	44.3 ± 7.0
Educational level, n (%)													
Illiterate and incomplete elementary school, 3 (8.8)	40.0 ± 22.9	56.7 ± 2.9	75.0 ± 33.1	66.7 ± 15.0	76.1 ± 18.4	91.7 ± 8.3	75.0 ± 5.0	81.3 ± 22.5	77.1 ± 29.5	33.3	11.0 ± 0.0	50.0 ± 43.3	74.2 ± 17.1
Complete elementary school, 5 (14.7)	76.0 ± 23.2	66.0 ± 14.7	87.5 ± 15.3	58.2 ± 12.0	60.5 ± 25.9	88.3 ± 17.3	57.0 ± 15.7	63.8 ± 13.1	62.5 ± 32.2		100.0 ± 0.0	80.0 ± 20.9	72.4 ± 12.2
Complete high school, 18 (52.9)	69.4 ± 17.2	68.9 ± 13.0	74.3 ± 22.1	78.7 ± 20.2	70.5 ± 21.7	88.9 ± 16.2	63.1 ± 21.8	70.1 ± 26.9	80.2 ± 19.8	56.3 ± 39.3	91.2 ± 19.6	62.5 ± 31.2	74.3 ± 17.5
Complete graduation, 8 (23.5)	56.6 ± 17.6	70.0 ± 10.4	67.2 ± 22.1	74.4 ± 25.6	78.4 ± 23.4	90.6 ± 17.5	56.9 ± 31.5	72.6 ± 30.4	78.9 ± 31.5	73.6 ± 22.6	93.7 ± 17.7	78.1 ± 28.1	76.3 ± 19.5
Monthly household incomes^b^, n (%)													
<197USD, 7 (21.2)	66.1 ± 19.9	**57.8 ± 6.7**	66.1 ± 27.7	59.4 ± 20.5	60.4 ± 19.7	79.8 ± 21.4	51.4 ± 19.9	58.0 ± 22.7	36.4 ± 18.2	16.7 ± 23.7	92.9 ± 18.9	50.0 ± 28.9	63.0 ± 11.9
197 to 590USD, 17 (51.5)	69.6 ± 22.5	**72.1 ± 14.1**	80.9 ± 20.8	77.2 ± 17.6	72.5 ± 23.4	93.6 ± 10.0	66.5 ± 23.5	76.5 ± 28.4	77.6 ± 29.6	75.0 ± 11.8	93.8 ± 17.1	72.1 ± 30.5	78.1 ± 17.2
>590USD, 9 (27.3)	55.3 ± 16.2	**67.2 ± 8.3**	70.8 ± 19.8	74.9 ± 23.9	76.4 ± 22.1	88.9 ± 18.2	59.4 ± 24.0	68.8 ± 28.5	86.1 ± 18.4	73.8 ± 21.7	94.4 ± 16.7	72.2 ± 31.7	75.8 ± 16.9
Marital status, n (%)													
Married, 19 (55.9)	65.8 ± 22.8	66.6 ± 13.3	75.0 ± 22.0	70.8 ± 26.7	65.2 ± 25.0	86.8 ± 18.3	56.3 ± 25.4	63.5 ± 29.4	70.7 ± 29.5	58.3 ± 33.6	88.9 ± 21.4	63.2 ± 30.5	70.3 ± 18.9
Single, 10 (29.4)	59.8 ± 17.8	70.5 ± 10.9	77.5 ± 20.2	81.7 ± 16.7	79.7 ± 18.4	91.7 ± 13.0	69.5 ± 19.9	83.1 ± 18.4	85.6 ± 15.9	66.7	100.0 ± 0.0	67.5 ± 33.4	80.6 ± 12.6
Divorced. 4 (11.8)	75.6 ± 15.3	70.0 ± 9.1	78.1 ± 18.8	66.5 ± 16.0	77.7 ± 9.9	95.8 ± 4.8	72.5 ± 17.1	76.6 ± 31.2	89.1 ± 12.9	83.3 ± 0.0	100.0 **± 0.0**	93.8 ± 12.5	81.8 ± 8.3
Widow. 1 (2.9)	52.5	50.0	25.0	75.0	81.3	91.7	45.0	62.5	62.5		100.0	50.0	64.3
Current housing situation, n (%)													
With spouse or family, 32 (94,1)	64.0 ± 20.1	66.9 ± 11.2	73.8 ± 22.5	73.1 ± 20.9	71.3 ± 21.8	90.1 ± 14.9	61.9 ± 22.9	69.7 ± 27.5	77.1 ± 25.1	63.6 ± 29.9	93.5 ± 17.0	66.4 ± 30.9	74.1 ± 16.6
Alone, 2 (5.9)	77.5 ± 31.9	80.0 ± 28.3	87.5 ± 0.0	81.3 ± 26.5	72.9 ± 38.3	79.2 ± 29.5	60.0 ± 28.3	87.5 ± 17.7	75.0 ± 35.4		100.0 ± 0.0	87.5 ± 17.7	81.1 ± 22.2
People sharing same habitation, n (%)													
Alone, 2 (5.9)	77.5 ± 31.8	80.0 ± 28.3	87.5 ± 0.0	81.3 ± 26.5	72.9 ± 38.3	79.2 ± 29.5	60.0 ± 28.3	87.5 ± 17.7	75.0 ± 35.3		100.0 ± 0.0	87.5 ± 17.7	81.1 ± 22.2
1 - 3 individuals, 22 (64.7)	61.6 ± 19.5	67.7 ± 11.0	70.5 ± 24.9	71.3 ± 21.2	69.9 ± 20.4	89.4 ± 15.7	58.6 ± 24.1	65.6 ± 28.4	75.6 ± 24.5	60.2 ± 31.7	92.9 ± 17.9	63.6 ± 29.6	72.1 ± 16.6
>4 individuals, 10 (39.4)	69.3 ± 21.3	65.0 ± 11.8	81.3 ± 14.7	77.1 ± 20.8	74.3 ± 25.6	91.7 ± 13.6	69.0 ± 19.0	78.7 ± 24.2	80.6 ± 27.2	79.2 ± 17.7	95.0 ± 15.8	72.5 ± 34.3	78.5 ± 16.5
Employment status, n (%)													
Unemployed + housewife, 10 (29,4)	66.5 ± 15.3	73.0 ± 10.6	76.2 ± 27.9	82.0 ± 18.7	80.5 ± 11.6	95.8 ± 4.4	71.5 ± 19.7	80.0 ± 25.8	87.5 ± 13.2		100.0 ± 0.0	77.5 ± 24.9	84.4 ± 11.7
Employed, 19 (55.9)	64.1 ± 23.3	65.8 ± 11.8	74.3 ± 17.4	71.9 ± 18.7	68.3 ± 22.2	86.0 ± 16.9	56.1 ± 21.5	65.5 ± 25.8	72.7 ± 25.0	61.5 ± 33.0	91.7 ± 19.2	60.5 ± 32.6	70.8 ± 15.7
Retired and away from work, 5 (14,6)	64.0 ± 21.8	64.0 ± 16.0	72.5 ± 29.9	63.3 ± 30.1	65.0 ± 35.2	90.0 ± 22.4	64.0 ± 29.7	72.5 ± 35.0	72.5 ± 39.9	69.4 ± 24.1	90.0 ± 22.4	75.0 ± 30.6	72.8 ± 25.1
Ocular manifestation, n (%)													
Anterior uveitis, 4 (11,8)	61.3 ± 32.8	73.8 ± 18.9	84.4 ± 15.7	81.3 ± 13.8	91.5 ± 7.5	**100.0 ± 0.0**	80.0 ± 8.2	98.4 ± 3.1	100.0 ± 0.0	83.3 ± 0.0	100.0 ± 0.0	**97.8 ± 12.5**	90.1 ± 4.8
Intermediate uveitis, 3 (8.8)	63.3 ± 20.1	58.3 ± 5.8	54.2 ± 31.5	54.2 ± 30.0	56.4 ± 24.1	**72.2 ± 19.2**	38.3 ± 31.8	60.4 ± 25.3	52.1 ± 34.4	62.5 ± 29.5	83.3 ± 28.9	**41.7 ± 28.9**	58.0 ± 19.6
Posterior uveitis, 17 (50,0)	62.3 ± 21.5	68.2 ± 10.6	76.5 ± 21.1	77.8 ± 21.1	72.6 ± 22.6	**91.1 ± 13.9**	65.0 ± 22.2	68.4 ± 26.6	78.3 ± 24.7	56.9 ± 37.4	94.1 ± 16.6	**75.0 ± 27.9**	76.5 ± 15.5
Panuveitis, 6 (16.7)	65.0 ± 14.9	65.0 ± 12.6	83.3 ± 15.1	69.7 ± 15.3	56.7 ± 22.0	**77.8 ± 18.0**	50.8 ± 18.3	66.7 ± 33.2	69.8 ± 26.6		90.0 ± 22.4	**41.7 ± 20.4**	66.4 ± 17.5
Other, 4 (11.8)	66.9 ± 20.2	70.0 ± 15.8	59.4 ± 25.8	68.5 ± 24.4	79.7 ± 12.3	**93.7 ± 4.2**	63.7 ± 20.6	67.2 ± 28.6	78.1 ± 15.7	66.7	100.0 ± 0.0	**68.7 ± 37.5**	74.8 ± 14.2
Laterality, n (%)													
Unilateral, 13 (38.2)	63.8 ± 22.4	69.2 ± 12.9	73.1 ± 23.9	78.8 ± 21.2	76.4 ± 18.1	92.9 ± 14.0	68.5 ± 19.4	79.8 ± 21.5	88.0 ± 14.6	58.3 ± 22.0	92.3 ± 18.8	69.2 ± 32.5	7.6 ± 16.1
Bilateral, 21 (61.8)	65.4 ± 19.8	66.7 ± 12.1	75.6 ± 21.5	70.4 ± 20.5	68.3 ± 24.3	87.3 - 16.4	57.6 ± 23.9	65.2 ± 29.2	70.2 ± 27.9	65.6 ± 33.4	95.0 ± 15.4	66.7 ± 29.9	71.9 ± 16.7
Visual status, n (%)													
Normal vision, 31 (91.2)	**62.3 ± 19.5**	**69.4 ± 11.4**	73.8 ± 22.4	**76.4 ± 19.0**	**75.5 ± 18.5**	91.4 ± 12.8	**64.4 ± 21.7**	**74.2 ± 25.7**	**80.6 ± 22.3**	70.0 ± 22.3	**96.7 ± 12.7**	69.4 ± 30.8	**76.9 ± 15.0**
Low vision, 3 (8.8)	**90.8 ± 8.8**	**50.0 ± 5.0**	83.3 ± 19.1	**44.4 ± 18.8**	**29.2 ± 7.2**	69.4 ± 29.3	**35.0 ± 15.0**	**35.4 ± 13.0**	**39.6 ± 25.3**	0	**66.7 ± 28.9**	50.0 ± 25.0	**48.9 ± 8.9**
Previous TB treatment, n (%)													
Yes, 7 (20,6)	57.5 ± 29.9	62.9 ± 11.1	82.1 ± 15.9	67.5 ± 26.0	66.8 ± 30.4	84.5 ± 22.3	51.4 ± 23.9	56.3 ± 29.8	67.0 ± 30.1	50.0 ± 70.7	92.9 ±18.9	67.9 ± 27.8	69.7 ± 17.1
No, 27 (79,4)	66.7 ± 17.6	68.9 ± 12.4	72.7 ± 23.3	75.2 ± 19.6	72.6 ± 20.1	90.7 ± 13.5	64.4 ± 22.0	74.5 ± 25.6	79.6 ± 23.6	66.7 ± 20.8	94.2 ± 16.3	67.6 ± 31.6	75.7 ± 16.6
Experienced prejudice because TB, n (%)													
Yes, 8 (23,5)	**79.4 ± 18.4**	61.9 ± 10.3	68.7 ± 26.7	**55.5 ± 26.6**	**55.1 ± 24.2**	82.3 ± 23.3	50.0 ± 28.2	53.9 ± 27.3	59.4 ± 33.7	46.7 ± 24.3	**81.3 ± 25.9**	71.9 ± 28.1	**63.5 ± 19.2**
No, 26 (76,5)	**60.3 ± 19.2**	69.4 ± 12.4	76.4 ± 20.7	**79.2 ± 15.4**	**76.4 ± 19.4**	91.7 ± 12.0	65.4 ± 19.9	76.0 ± 25.4	82.5 ± 19.5	75.0 ± 22.0	**98.0 ± 10.0**	66.3 ± 31.6	**77.9 ± 14.5**
Concurrent uveitis treatment, n (%)													
Yes, 12 (35,3)	**60.2 ± 22.2**	**70.7 ± 12.9**	79.5 ± 21.3	**82.1 ± 16.6**	**79.0 ± 19.7**	**95.5 ± 9.2**	**70.2 ± 19.1**	**79.8 ± 23.6**	**83.2 ± 23.6**	70.8 ± 25.7	97.7 ± 10.7	**79.6 ± 23.9**	**81.6 ± 12.6**
No, 22 (64,7)	**73.1 ± 14.1**	**62.1 ± 8.9**	65.6 ± 21.4	**58.1 ± 19.2**	**57.5 ± 20.3**	**78.5 ± 19.0**	**46.3 ± 20.9**	**54.2 ± 26.0**	**65.6 ± 24.6**	55.0 ± 35.2	86.4 ± 23.4	**45.8 ± 29.8**	**61.5 ± 15.4**

TB= tuberculosis. ^a^n=11;

b Monthly household income not informed by one participant. Statistical analyses were performed using the Mann-Whittney U-Test (bold values denote statistical significance at the p<0.05 level).
